# Plasticity and Anxiety

**DOI:** 10.1155/2007/75617

**Published:** 2007-07-30

**Authors:** Patrice Venault, Georges Chapouthier

**Affiliations:** UMR CNRS 7593, Groupe Hospitalier Pitie-Salpetriere, 91 Boulevard de l'Hôpital, 75634 Paris Cedex 13, France

Anxiety is a very broad behavioural trait, helping animals to cope with dangerous environmental situations. As anxiety is linked to other emotional processes and to cognitive functions such as learning and memory, it involves a number of cerebral structures and brain transmitter systems, thereby giving rise to a high degree of plasticity. Within the limited space of a single issue, it is obviously impossible to cover all aspects of anxiety processes occurring in the brain. By including both extensive reviews and articles reporting on experiments, the present issue wishes to present clearly different approaches to this ubiquitous behaviour trait.

The reviews start with the extensive review by D. M. Diamond et al. covering the effects of stress on LTP in the hippocampus, amygdala, and prefrontal cortex. Here the authors are presenting challenging new hypotheses on putative “temporal dynamics” of plasticity, involving an “activational” period, followed by a consolidation process and making it possible to recall traumatic memories. Another approach, which is complementary to this model, focuses on the relationship between stress and memory processes and is reviewed by C. Sandi and M. T. Pinelo-Nava who made a classification according to five factors: (1) the source of the stress, (2) the duration, (3) the intensity, (4) the timing, that is, in relation to the memory phase, and (5) the type of learning experience; a clear analysis has been made of the effects of stress on memory and of the main neurobiological mechanisms involved. I. Akirav and M. Maroun have investigated the role of the medial prefrontal cortex-amygdala circuit in the effects of stress on fear and the extinction of fear, and the key role played by the GABA transmitter system.

A. V. Kalueff has conducted an extensive review covering neurogenetic, neurochemical, and behavioural aspects, and the essential relationship between anxiety and memory, that is, between emotional and cognitive factors in the functioning of the brain. A strong relationship found in both human and animals was investigated by A.V. Kalueff and D. L. Murphy: the relationship between anxiety, depression, and stress-related disorders; such stress-related disorders can also lead to cognitive dysfunctions affecting learning and memory. The importance of hippocampal neurogenesis in these phenomena and their link with antidepressant treatment were studied by E. Paizanis et al. The involvement of the endocannabinoid system in the regulation of anxiety and in brain plasticity of emotional states is the subject of the analysis by M.-P. Viveros et al. In patients with early onset Alzheimer's disease (under the age of 65), apolipoprotein E4 is a risk factor. In rodents, this same protein plays a role in the regulation of anxiety; this is reviewed by J. Raber. C. Belzung and P. Philippot chose a phylogenetic approach ranging from specific reactions to danger in simple organisms to more elaborate physiological and behavioural responses in “higher” animals, and culminating with autonoetic consciousness of anxiety in great apes and humans.

Experimental data provide evidence for key arguments in the reviews. G. Legradi et al. studied rats and highlighted the action of pituitary adenylate cyclase activating polypeptide (PACAP) when administered in the central nucleus of the amygdala; they have shown that PACAP induces reorganization of stress-coping behaviour, with a shift from an active mode (burying) to a passive mode (withdrawal or immobility). V. Brinks et al. used mice to study the involvement of high affinity mineralocorticoid receptors and low affinity glucocorticoid receptors in the regulation of emotion and cognition in mice. Increased corticosterone concentrations and the gradual switch from mineralocorticoid to glucocorticoid receptors produced a “strong emotional arousal at the expense of cognitive performance.” By studying two strains of mice in tests classically used to assess anxiety and comparing the results through a principal component analysis study, Y. Clement et al. identified four essential behaviour patterns: novelty-induced anxiety, general activity, exploratory behaviour, and decision making. In clinical practice with humans, L. Carmilo-Granado et al. found evidence for a new treatment of anxiety in cases of arachnophobia. Patients were shown computer images, not of spiders but of objects with spider-like features (e.g. the Atomium in Brussels), and the authors managed to induce a sharp reduction in the symptoms of arachnophobia.

As can only be expected, and as can be seen with all the data and arguments presented here, this issue presenting a variety of approaches will not give a clear-cut answer to the question of the plasticity of anxiety, but instead will open a number of new paths for future research and discovery.

*Patrice Venault* *Patrice Venault*

*Georges Chapouthier* *Georges Chapouthier*

